# Supplementary material for: Humor in parenting: Does it have a role?
Source: PLoS One. 2024 Jul 17;19(7):e0306311. doi: 10.1371/journal.pone.0306311 (PMC11253938; doi:10.1371/journal.pone.0306311)
Supplement: Attachment — Submitted filename: renamed_3078e.docx [file pone.0306311.s001.docx]

We appreciate and agree with the reviewers’ recommendations for improving this manuscript. Our efforts to address them are as follows. (*page and line numbers refer to the CLEAN version*)

| RESPONSES TO REVIEWER COMMENTS | | |
| --- | --- | --- |
| Reviewer Comments | How addressed | Page  (line) |
| REVIEWER #1. |  |  |
| 1. On pag. 5, lines 99-100, the authors give a philosophical and psychological overview of the main theories on humour, but only Morreall's philosophical work is mentioned work is mentioned (line 106). I would suggest adding Martin's book 'The psychology of humour. An integrative approach' (2018), for a more complete picture. | We have augmented this section. | 5  (101-112) |
| 2. It is not clear which statistical test was used for the results presented in Tables 2 and 3. I assume they are Pearson correlations, but it would be better to make this explicit. Furthermore, the data in Tables 2 and 3 are not easy to interpret compared to what is clearly stated in the text. I would suggest modifying accordingly. | We have 1) clarified that we used an exact t-test, 2) provided the exact number of participants for each cell, and 3) provided row percentages. | 23  (585-586) |
| REVIEWER 2 |  |  |
| 3. My primary concern with this paper is the oversimplification of the term "humor." There is a substantial body of literature that investigates the differentiation of humor types, such as Humor Styles (Martin et al., 2003) and Comic Styles (Ruch et al., 2018). | We have significantly bolstered the introduction by providing more conceptual background about research on the nature, forms, styles, and outcomes of humor. | 5  (101-112) |
| 4. In the introduction, I expected the authors to delve deeper into research exploring the relationship between humor and attachment, particularly addressing the challenges young children face in detecting and understanding irony and sarcasm.. | We now touch on humor’s relationship with secure attachment. However, because studies show only weak associations between adaptive humor and secure attachment, we do not delve more deeply into this connection. | 7  (147-150) |
| 5. Moreover, the introduction could benefit from a clearer statement of the paper's objectives and research questions to guide the reader | We now explicitly state the study’s exploratory goals. | 4&5  (96-99) |
| 6. It would be helpful to provide clear definitions or explanations for terms such as "cognitive flexibility," "frameshifts," and "psychological distance" to ensure that the reader understands these concepts in the context of the paper | We have now provided additional clarification of these terms. | 4&6  (90-92)  (133-134) (136-137) |
| 76. The literature review is not comprehensive and do not cover a range of theories and studies related to humor and parenting, especially related to attachment theory. I therefore recommend the authors to cite more literature in this field | We now mention and cite literature involving both theories and studies related to humor and parenting. | 5  (101-109)  7  (148-150) |
| 8. The paper discusses various theories of humor but could provide a more critical analysis of how these theories apply to parenting. For example, the paper mentions that incongruity-based humor contradicts the need for consistency in parenting, but it could delve deeper into how parents can balance humor and consistency effectively. | Though it is beyond the scope of this exploratory pilot study to delve deeply into the potential applications of theory to practice, we now briefly discuss connections between incongruity and conflict management. | 7  (153-154) |
| 9. The paper identifies a significant gap in empirical research on humor as a parenting strategy. However, the paper could strengthen its argument by discussing why this gap exists and why it is important to address it. | While it is beyond the scope of this exploratory pilot study to speculate on why the gap exists, we now more clearly identify the gap and the potential value of filling it. | 4&5  (96-99) |
| 10. The conclusion summarizes the key points of the paper but could be strengthened by revisiting the research questions and summarizing the potential implications of the study's findings. Additionally, the paper could provide more specific recommendations for future research to address the identified gaps and limitations. | We have now expanded the discussion to address these recommendations. | 14  (334-339) |
|  |  |  |
|  |  |  |
